# Emotional development in eating disorders: A qualitative metasynthesis

**DOI:** 10.1002/cpp.2365

**Published:** 2019-04-25

**Authors:** Ziporah B. Henderson, John R.E. Fox, Penny Trayner, Anja Wittkowski

**Affiliations:** ^1^ Division of Psychology and Mental Health, School of Health Sciences University of Manchester Manchester UK; ^2^ School of Psychology, Cardiff University Cardiff UK; ^3^ Department of Clinical Psychology, Greater Manchester Mental Health NHS Foundation Trust Manchester UK

**Keywords:** eating disorders, emotions, qualitative research, metasynthesis review, social, emotional regulation

## Abstract

Emotions are considered to be an important feature in eating disorders. The present study aimed to conduct a systematic review and metasynthesis of qualitative studies, which considered the role of emotions in eating disorders in order to gain further insight on how these individuals experience various emotions and the strategies they use to manage them. Databases including Web of Science, PsychInfo, EMBASE, Medline, and the Cochrane library were searched for qualitative studies. The search identified 16 relevant studies. Meta‐ethnography was used to synthesize the data, which involved identifying the key findings and concepts of the studies and creating metaphors. The synthesis involved reciprocal translations and lines of argument approaches being applied to the present data. Results of the synthesis identified four second‐order themes and one third‐order theme relating to the emotional experiences of such individuals. The second‐order themes were (a) negative emotional environments, (b) interpersonal vulnerability, (c) the experience of negative emotions in social contexts, and (d) the management of emotions. The third‐order theme was the emotional self within a social environment. This is the first metasynthesis on emotions and eating disorders, and our synthesis highlights the important role that emotions play in the development and maintenance of eating disorders. Our model demonstrates how poor emotional development whilst growing up results in development of poor socioemotional bonds and the inability to handle negative emotions. The most significant finding of the review is that individuals use their eating disorder to manage negative emotions.

**Key Practitioner Message**

- Individuals with eating disorders have poor emotional skills in terms of managing and expressing their emotions socially, which may be due to being exposed to negative emotional environments whilst growing up. Therapeutic interventions need to explore the presence of these issues.
- Poor emotional skills appear to result in the avoidance of social and interpersonal relationships with family and peers amongst individuals with eating disorders.
- Eating disorder symptoms and behaviours, including food restriction and purging, are used as a means of managing negative emotions both in interpersonal and intrapersonal situations.
- Our understanding and treatment of individuals with eating disorders needs to consider the different aspects of the individual's social, interpersonal, and emotional well‐being.

## 1 INTRODUCTION

Eating disorders, including anorexia nervosa (AN), bulimia nervosa (BN), and eating disorders not otherwise specified (EDNOS), are amongst the most serious of mental illnesses (see APA, 2000; Nielsen, 2001; Papadopoulos, Ekbom, Brandt, & Ekselius, 2009). EDNOS (DSM‐IV‐TR, APA, 2000), rather than other specified feeding or eating disorder (ED; DSM‐V, APA, 2013), is being referred to in the current review as the studies reviewed included participants diagnosed with an ED based on the older classification system. These disorders involve the common concerns about gaining weight and associated behaviours to lose weight including food restriction and purging (see APA, 2000). Comorbidity with other psychiatric illnesses is very common including anxiety and depression (O'Brien & Vincent, 2003; Steinhausen, 2002). Patients with AN are often resistant to change and recover with there being up to a 50% relapse rate (see Fairburn, 2005; Pike, 1998). This is due to the lack of motivation to change because EDs often play a functional role (Cockell, Geller, & Linden, 2002; Fairburn, Shafran, & Cooper, 1999; Geller, Williams, & Srikameswaran, 2001; Serpell, Treasure, Teasdale, & Sullivan, 1999; Vitousek, Watson, & Wilson, 1998), with early modern theorists suggesting that AN serves as a means of gaining control during a time when a perceived lack of control is common (see Bruch, 1978; Crisp, 1997).

The present review explored the qualitative literature on emotions and EDs. As this review demonstrates how individuals with EDs experience and manage their emotions, there will be a consideration of emotion regulation theories including the SPAARS model of EDs (SPAARS‐ED an acronym for the Schematic, Propositional, Associative, Analogical Representations emotions may have; Fox & Power, 2009; Power & Dalgleish, 2008), the cognitive–interpersonal maintenance model of AN (Schmidt & Treasure, 2006; Treasure & Schmidt, 2013), and the integrative neuroscience model of AN (INTEGRATE‐AN; Hatch et al., 2010).

The role of emotions and emotional regulation in EDs, and particularly AN, has long been considered an important feature in the aetiology and maintenance of the disorder (Bruch, 1973), but this has not been prioritized in research and treatment programmes until recently (see Fox, Federici, & Power, 2012a; Haynos & Fruzzetti, 2011). Emotions are comprised of five different categories, namely, happiness, sadness, anger, fear, and disgust, with more complex emotions being built on a combination of these basic categories (Ekman, 1982, 1992a, 1992b). Emotions play a key role in everyday functioning in that they facilitate understanding of events and responses to them based on the individual's appraisals (Fox et al., 2012a; Oatley & Johnson‐Laird, 1987; Power & Dalgleish, 1997; Zajonc, 1980).

Emotion regulation theories have been used to help understand the role of emotional functioning in EDs (Gross, 1998, 2002). Emotion regulation relates to processes individuals engage in in order to influence the emotions they experience, when the emotions are experienced, the degree to which they are experienced and whether they are expressed (Gross, 1998; Sloan & Kring, 2007). Studies have illustrated relationships between deficits in emotion regulation strategies and numerous psychiatric disorders and EDs (Aldao, Nolen‐Hoeksema, & Schweizer, 2010; Danner, Evers, Stok, van Elburg, & de Ridder, 2012; Wolgast, Lundh, & Viborg, 2013). ED symptoms including food restriction, purging, and binge eating serve as a means of “escape” from negative emotions due to their numbing effect on negative arousal (Haynos & Fruzzetti, 2011). Thus, patients with EDs may be using their ED symptoms and related behaviours as a means of regulating their emotions by directing them towards the body (see Aldao et al., 2010; Danner et al., 2012).

The SPAARS‐ED (Fox & Power, 2009; Power & Dalgleish, 2008) builds on modern theories on emotions and the research literature suggesting the role of emotions in EDs (Fox & Power, 2009). The SPAARS model suggests that emotions can be categorized into five basic domains: happiness, sadness, anger, fear, and disgust (Fox & Power, 2009). A central feature of the model is that individuals learn which emotions are acceptable for them to express, that is, ego‐syntonic, and which ones are considered unacceptable for them to express, that is, ego‐dystonic, and as a result are inhibited by the individual (Fox & Power, 2009; Power & Dalgleish, 1997). As part of development and growing up, individuals learn which emotions are “acceptable” or not acceptable to express; for example, individuals learn that anger is a dangerous emotion (see Fox, 2009; Fox & Power, 2009). Emotions, which are considered to be ego‐dystonic and potentially “dangerous” for the individual to express, due to them being perceived as negative and having the potential for them to cause rejection, are suppressed using ED behaviours and are directed towards the body (Fox & Power, 2009).

By contrast, the cognitive–interpersonal maintenance model of AN (Schmidt & Treasure, 2006; Treasure & Schmidt, 2013) proposes that certain factors predispose individuals to AN. In addition, the individual's engagement in food restriction and the effects of starvation make these risk factors become more prominent in their influence on the individuals behaviours and are responsible for the maintenance of the disorder (Schmidt & Treasure, 2006; Sternheim et al., 2012; Treasure & Schmidt, 2013). The model proposes that emotional avoidance through the avoidance of social interactions is also evident amongst individuals prior to the onset of an ED. Engaging in ED behaviours and the effects of starvation further exacerbates these social and emotional skills and therefore become maintenance factors for the disorder (Schmidt & Treasure, 2006; Treasure & Schmidt, 2013).

In addition, the integrative neuroscience model of AN (INTEGRATE‐AN; Hatch et al., 2010), a stress‐diathesis model linking emotions to ED behaviours in a temporal sequence (Hatch et al., 2010), suggests that certain genetic and constitutional risk factors, including puberty changes (see Kaye et al., 2005; Kaye, Frank, Bailer, & Henry, 2005; Sisk & Foster, 2004; Treasure, 2007), make individuals more sensitive to negative emotional cues and that this coupled with a stressful life event; for example, starting high school or death of a close one can trigger AN (Hatch et al., 2010). The hypersensitivity to negative emotions also results in a feeling of loss of control, and as a result, such individuals engage in food restriction, which serves as a maladaptive strategy for regulating their emotions (due to the numbing effect food deprivation has on emotions). This “starvation syndrome” exacerbates predispositional factors and results in the maintenance of the ED (see Hatch et al., 2010).

The above research and models demonstrate that emotions play an important role in EDs. However, the evidence referred to has been drawn primarily from quantitative research. In recent years, there has been some qualitative research in EDs, with particular consideration on the role of emotions in EDs. However, to date, there has been no systematic review of these qualitative studies. The aim of the present review is to conduct a metasynthesis of studies considering emotions in EDs in order to gain an in depth account on how individuals with EDs experience various emotions and the strategies they use to manage them.

## 2 METHOD

### 2.1 Systematic literature search

The search involved identifying published journal articles, which considered the role of emotions in EDs via electronic searches of databases including, Web of Science, PsychInfo, EMBASE, Medline, and the Cochrane library. The searches were not limited by date because very few articles prior to the 1980s were found. The systematic searches were updated in January 2019. In accordance with the guidelines from the Evidence for Policy and Practice Centre (EPPI‐Centre, 2008), the search terms used related to three different categories relevant to this review: (a) EDs, (b) emotions, and (c) qualitative research (see Table 1). Search terms from each of the three categories were combined using “OR” and “AND,” and truncation was used for the key terms. In addition, the reference lists of any full text articles that were obtained were also searched for any potentially relevant articles. The systematic literature search was conducted in accordance with PRISMA guidelines (Moher, Liberati, Tetzlaff & Altman, 2009).

**Table 1:** Search terms used in the systematic search of the electronic databases

Word relating to eating disorder	Search terms used
Eating disorders	Eating disorder*
Eating behaviours	Eat*
Anorexia nervosa	Anore*
Bulimia nervosa	Bulimi*
Eating difficulties	Eating difficult*
Food restriction	Food restriction
Binge eating	Bing*
Weight concerns	weight
Dieting behaviours	Diet*
Words relating to emotions	Search terms used
Emotion regulation	Emotion regulation
Emotions	Emotion*
Self‐conscious emotions	Self‐conscious affect* and self‐conscious emotion*
Shame	Shame
Pride	Pride
Embarrassment	Embarrass*
Guilt	Guilt*
Social cognitions	Social cognition*
Sadness	Sad*
Anger	Anger*
Fear	Fear*
Disgust	Disgust*
Happiness	Happy and happi*
Words relating to qualitative research	Search terms used
Qualitative research	Qualitative research and qualitative stud*
Phenomenology	Phenomenolog*
Grounded theory	Grounded theor* and grounded stud*
Ethnography	Ethnograph*
Ethnomethodology	Ethnomethodolog*
Hermeneutic	Hermeneutic
Content analysis	Content analysis
Focus groups	Focus group*
Thematic analysis	Thematic* and theme*
Discourse analysis	Discourse analysis

### 2.2 Inclusion or exclusion criteria

The following inclusion criteria were used: (a) studies had to be in English; (b) participants had to be recruited from a known ED group, that is, AN, BN, or EDNOS; (c) the studies had to have used a qualitative research methodology; and (d) a main focus of the study had to be on emotions.

The exclusion criteria were (a) studies that did not use a qualitative research methodology, (b) studies that did not use a clinical sample of ED participants, (c) studies that did not focus on emotions or if there was not a substantial amount of information on emotions in the results, and (d) studies that did not focus on emotions in the aetiology of an ED but on recovery.

### 2.3 Critical appraisal

In order to critically appraise the studies, a checklist of 10 criteria drawn from two established checklists (CASP, 2006; Walsh & Downe, 2006) was used. It included concerns relating to (a) the aims of the study, (b) methodology, (c) data collection, (d) strategy used for sampling, (e) process of analysis, (f) results, (g) ethical issues, (h) reflexivity, (i) consideration of the transferability of the findings, and (j) whether the context of the research was taken into account.

If a criterion was not considered at all in the study, it received a score of “0,” if it partly met the criteria or did not use the best means of fulfilling the criteria, it received a score of “0.5,” and if it fully met the criteria, it received a score of “1.” The studies were critically appraised by the first author (Z. H.), and another author (J. F.) co‐rated a sample; there was 100% agreement between both raters.

### 2.4 Data synthesis

A data extraction table was developed (see Table 2). Authors, title, study focus, location, sample, diagnosis, data collection, and analysis were extracted from the studies included. Noblit and Hare's (1988) metaethnography approach was used to synthesize the data. This method was chosen because it is designed to be an interpretive means of data synthesis through comparing findings from several qualitative studies in a systematic way so that an overall translation can be made (Noblit & Hare, 1988). The first author (Z. H.) read the studies several times to gain familiarity with all the data and then made brief notes that highlighted the key findings in each of the studies, and these were the first‐order translations. The first‐order translations and notes were then reviewed several times to gain further familiarity and consider in depth the relationships between the concepts from each of the studies. Findings from the different studies were merged when they overlapped or added as a new concept (in the synthesis) when they demonstrated a new aspect for the synthesis. Reciprocal translations and lines of argument approaches were most relevant for the present data. The first author (Z. H.) then created metaphors and themes, which encapsulated the main findings of the synthesis, with these being the second‐order themes and the fundamental part of this synthesis. The third‐order theme builds on these second‐order themes and demonstrates a more advanced way of conceptualizing the results in this review. At each stage of the synthesis process, the first author consulted another author (J. F.) to check if there was a general consensus of opinion, which was then agreed by all of the authors.

**Table 2:** Characteristics of the included studies in chronological order

Study	Authors and title	Study focus	Location	Sample	Diagnosis	Data collection	Data analysis	Quality
1	Jeppson, Richards, Hardman, and Mac Granley (2003): Binge and purge processes in bulimia nervosa: A qualitative investigation	Function of the binge–purge cycle	USA	8 women, 20–39 years	7 inpatients—1 outpatient, all BN, illness duration 15 months to 14 years	Interviews, ~60 min	Emergent data analysis (Glaser & Strauss, 1967; Lincoln & Guba, 1985)	8.5
2	Wasson (2003): A qualitative investigation of the relapse experiences of women with bulimia nervosa	Emotional and interpersonal factors associated with relapse amongst individuals with BN	USA	26 women, 20–59 years	All had BN, all been in recovery state for minimum of 6 months	Focus groups (~3 hr) and interviews (~90 min)	Constant comparative method (Crabtree & Miller, 1992; Patton, 1990)	6
3	Nordbo, Espeset, Gulliksen, Skarderud, and Holte (2006): The meaning of self‐starvation: qualitative study of patients' perception of anorexia nervosa	Psychological meaning of AN according to patients	Norway	18 women, 20–34 years (mean 25.5)	14 outpatients—4 inpatients, all AN, average duration 10 years (range 1–22 years), average treatment 6 years (range 1/2–14 years), lowest BMI during illness 12.9 (range 8–16), 12 satisfied AN criteria at interview	Semiopen “Experience interview” (Holte, 2000)	Phenemonological design (Moustakas, 1994) with Grounded theory (Strauss & Corbin, 1998)	7.5
4	Skarderud (2007): Shame and pride in anorexia nervosa: A qualitative descriptive study	The role of shame in AN	Norway	13 women, 16–39 years	8 restrictive AN—5 bulimic AN, illness duration 1–19 years, BMI range 10.8–17.6, all had received min 6 months treatment‐private psychotherapy	Semistructured interviews	Method of analysis not clearly stated	7
5	McNamara, Chur‐Hansen, and Hay (2008): Emotional responses to food in adults with an eating disorder: a qualitative exploration	ED patients' emotional responses to food exposure	Australia	10 women, 18–41 years (mean 29.1)	Community sample, 5 BN—3 EDNOS—2 AN, BMI range 17.51–40.75 (mean BMI 24.49	Semistructured interviews (35–60 min)	Framework Approach (Pope, Ziebland, & Mays, 2000)	6
6	Fox (2009): A qualitative exploration of the perception of emotions in anorexia nervosa: A basic emotion and developmental perspective	The role emotions play in AN	UK	11 women, 19–51 years	5 inpatients—6 outpatients, 5 restrictive AN, 6 bulimic AN, BMI range 13.3–20.1	Interviews (60 min)	Grounded theory (Charmaz, 2006; Glaser & Strauss, 1967)	9
7	Kyriacou, Easter, and Tchanturia (2009): Comparing views of patients, parents, and clinicians on emotions in anorexia: A qualitative study	The role of emotions in AN from the different perspectives of patients, parents, and clinicians	UK	6 women, 20–36 years (mean −26.8)	All inpatients, 4 restricting AN, and 2 binge–purge AN, 4–22 years illness duration (mean 10.7 years), BMI range 12–16.10 (mean BMI 14.3)	Focus groups	Thematic analysis (Braun & Clarke, 2006)	7
8	Rortveit, Astrom, and Severinsson (2009): Experiences of guilt as a mother in the context of eating difficulties	The experiences of mothers with EDs	Norway	8 women, 23–48 years	7 outpatients—1 inpatient, illness duration 10–30 years, BMI range 14.9–33.1, 2 nearly recovered	Interviews, 30–60 min	A hermeneutic approach (Gadamer, 1975) and qualitative content analysis (Graneheim & Lundman, 2004)	7
9	Rortveit, Vevatne, and Severinsson (2009): Balancing between mental vulnerability and strength in daily life when suffering from eating difficulties	Emotional and other psychological issues amongst women with EDs	Norway	5 women, 28–48 years (mean −35.8)	4 inpatients—1 outpatient, 10–14 years illness duration (mean 11.4 years), BMI range 14.9–33.1 (mean BMI 22.18)	Focus groups 30‐min general conversation, 40‐min art activity, 50‐min verbal reflection	Content analysis using hermeneutic approach (Gadamer, 1975)	7.5
10	Rortveit, Astrom, and Severinsson (2010): The meaning of guilt and shame: A qualitative study of mothers who suffer from eating difficulties	Guilt and shame in mothers with EDs	Norway	8 women, 23–48 years	7 outpatients—1 inpatient, ED subtypes not distinguished between, 10–30 years duration, BMI range 14.9–33.1	Interviews, 30–60 min	Content analysis (Graneheim & Lundman, 2004)	7
11	Tierney and Fox (2010): Living with the ‘anorexic voice’: A thematic analysis	The inner voice amongst individuals with AN	UK	21 women, mean age 22.1 years	Recruited from self‐help groups, all AN, average current BMI 17.1, 12 participants displayed AN at time of study	Poems, letters, and descriptive narratives about the inner voice	Thematic analysis (Braun & Clarke, 2006)	6
12	Espeset, Gulliksen, Nordbo, Skarderud, and Holte (2012): The link between negative emotions and eating disorder behaviour in patients with anorexia nervosa	Link between negative emotions and ED behaviours	Norway	14 women, 19–39 years (mean 29.1)	6 outpatients—8 inpatients, 6 restrictive AN—8 bulimic AN, average AN duration 10 years (range 3–25 years), Lowest BMI 14.6 (range 12–16), current BMI 17.5 (range 14.2–23.1)	Interviews, 75–120 min	Grounded theory (Corbin & Strauss, 2008)	7.5
13	Koruth, Nevison, and Schwannauer (2012): A grounded theory exploration of the onset of anorexia in adolescence	Emotions and how they are related to onset of AN	UK	8 participants, 7 women, and 1 man, 13–17 years	Inpatients and outpatients, all AN, 3–12 months illness duration	Interviews	Grounded theory (Charmaz, 2006; Glaser & Strauss, 1967; Strauss & Corbin, 1990)	5
14	Williams and Reid (2012): It's like there are two people in my head: A phenomenological exploration of anorexia nervosa and its relationship to the self	The experiences of individuals with AN with a focus on the function of AN with particular reference to emotions and the anorexic voice	UK	14 participants, 12 women, and 2 men, 21–50 years (mean 27)	Recruited from prorecovery websites, 8 restricting AN and 6 EDNOS, participants recruited online from USA (*n* = 8), UK (*n* = 4), Canada (*n* = 1), and Australia (*n* = 1)	Online focus group and e‐interviews	Interpretative phenomenological analysis (IPA, Smith, Jarman, & Osborn, 1999)	7.5
15	Faija, Tierney, Gooding, Peters, and Fox (2017): The role of pride in women with anorexia nervosa: A grounded theory study	The role pride plays in the aetiology and maintenance of anorexia nervosa	UK	21 women, 18–61 years (mean 29.67)	8 inpatients and 3 day care patients recruited from an eating disorder unit in the North West of England and 10 recruited from the U.K. charity Beat, all AN, 1 week to 43 years illness duration	Semistructured interviews (~60 min)	Grounded theory (Charmaz, 2006)	8.5
16	Rance, Clarke, and Moller (2017): The anorexia nervosa experience: Shame, solitude and salvation	The role anorexia nervosa plays in relation to the feelings and behaviours of sufferers	UK	12 women, 18–50 (mean 30.67)	Recruited from the U.K. charity Beat, 11 had AN diagnosis and 1 long behavioural history of dietary restriction, 13.3 years illness duration	Semistructured interviews (~59–103 min)	Thematic analysis (Braun & Clarke, 2006)	7.5

*Note*. AN: anorexia nervosa; BMI: body mass index; BN: bulimia nervosa; ED: eating disorder; EDNOS: eating disorders not otherwise specified.

## 3 RESULTS

**Figure 1:**
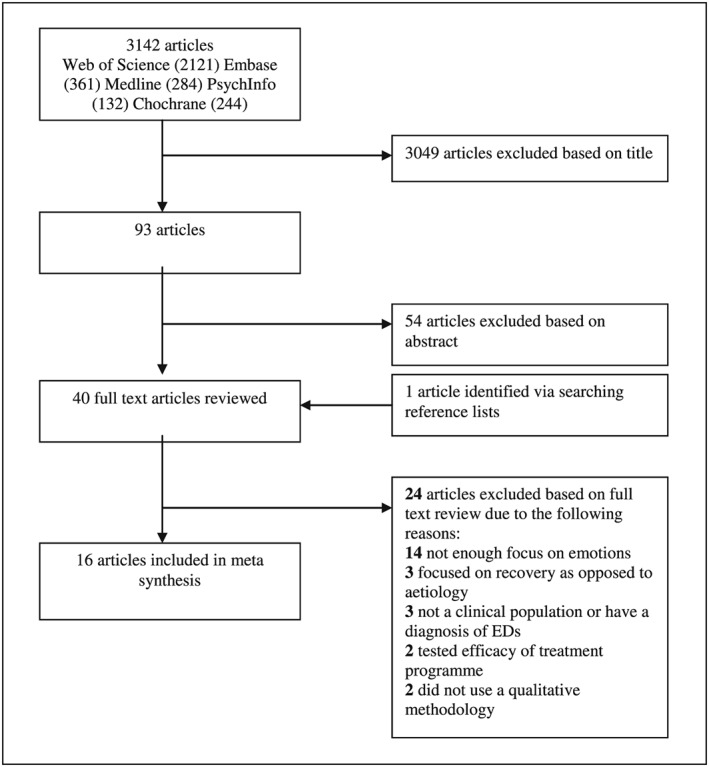
Flow chart illustrating results of the search strategy and the inclusion and exclusion process

The systematic searches identified a total of 3,142 articles. Of these articles 16 met all the inclusion criteria and were included in the metasynthesis. The majority of the articles were excluded due to the fact that they did not consider emotions, did not involve samples with EDs, or did not use a qualitative research methodology. Details of the process for the inclusion and exclusion of articles are identified in Figure 1.

The total number of participants included across all the studies was 203, including three male participants. The age range of participants was 13–61 years. Participants experienced either AN (*n* = 140, 69%), BN (*n* = 39, 19.2%), or EDNOS (*n* = 3, 1.5%). For some participants, their diagnosis was not recorded or stated, although they were identified via ED units (*n* = 21, 10.3%). Research demonstrates that amongst individuals with a diagnosis of an ED, EDNOS is the most common diagnosis followed by BN and then AN (see Fairburn & Bohn, 2005). This review suggests that research in this area has mainly focused on AN, as the majority of participants included in this review had a diagnosis of AN. The studies were conducted either in the United Kingdom, the United States, Australia, or Norway. The qualitative methodologies utilized by the studies included interviews and focus groups, and a range of analysis techniques were also used (see Table 2).

The quality ratings ranged from 5–9 (with a maximum of 10). One of the main flaws identified with the studies was that the samples were not assessed for the purpose of the study itself and relied on clinical notes to identify participants' ED, with the exception of two studies (Faija et al., 2017; Williams & Reid, 2012) that used the Eating Disorder Examination‐Questionnaire (Fairburn & Beglin, 1994). Many studies lacked researcher reflexivity and studies often lacked a consideration of the context in which the study was conducted. However, no studies were excluded on the basis of quality rating because none were considered to have any major methodological weaknesses and all provided significant information on the role of emotions in EDs. Details of the 16 included studies are presented in Table 2 in chronological order.

### 3.1 Synthesis

**Figure 2:**
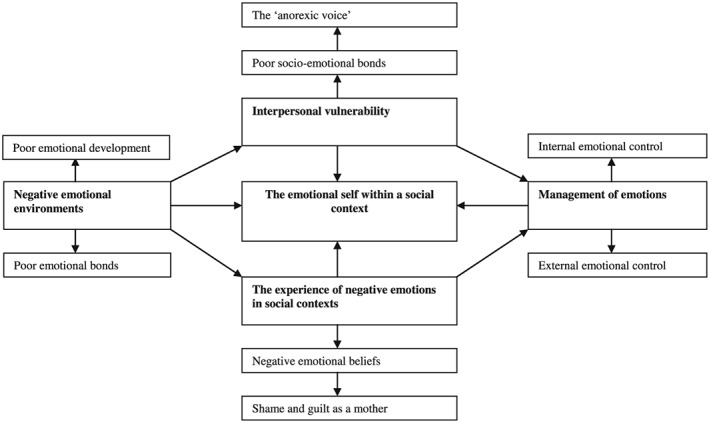
Model illustrating the main themes and the relationship between them

The results of the metasynthesis identified four second‐order themes and one third‐order theme, with the second‐order themes each consisting of several subthemes. The themes were “negative emotional environments,” “interpersonal vulnerability,” “the experience of negative emotions in social contexts,” and “management of emotions.” The third‐order theme that emerged from the data demonstrated “the emotional self within a social context.” These themes and how they relate to each other is demonstrated in Figure 2.

### 3.2 Theme 1: Negative emotional environments

This theme demonstrates how individuals with EDs experienced poor emotional environments during their childhood, which were often characterized by a lack of emotional expression or the presence of too many negative emotions. This resulted in the development of poor emotional skills. The subthemes, which will be considered here, are those of (a) poor emotional development and (b) poor emotional bonds.

#### 3.2.1 Poor emotional development

Individuals with an ED described how in general they found it difficult to identify, describe, and tolerate their own emotions. They appeared to experience emotions, and particularly negative emotions, as overwhelming (Fox, 2009; Koruth et al., 2012; Kyriacou et al., 2009; Rortveit, Vevatne, & Severinsson, 2009; Wasson, 2003). The negative emotions experienced as overwhelming included anger, sadness, worry, loneliness, depression, frustration, and guilt. Some participants highlighted how going through an episode of experiencing any of these emotions was one of their hardest challenges (Fox, 2009; Koruth et al., 2012; Rortveit, Vevatne, & Severinsson, 2009; Wasson, 2003). They also lacked emotional balance, with their emotions quickly changing from one extreme to another, resulting in emotional confusion (Kyriacou et al., 2009; Rortveit, Vevatne, & Severinsson, 2009).

… And then suddenly something happens and it's like AAGGH, and then it's back to absolutely nothing again. So I think if I could identify it at the time, it wouldn't explode, and I wouldn't be numb, you know. I could find something in between, that would be helpful …. (Kyriacou et al., 2009, p. 850)

Participants expressed how they struggled to describe and express their emotions. The data revealed two possible reasons for this (Fox, 2009; Kyriacou et al., 2009). First, participants failed to express their emotions due to the negative beliefs they held about expressing negative emotions, including the belief that emotions are frightening, a bad thing, and demonstrates weakness (Fox, 2009; Kyriacou et al., 2009). Second, participants failed to express their emotions due to the fact that they felt they lacked the ability to do so (Fox, 2009; Kyriacou et al., 2009). They either felt they had no one to confide in or they experienced confusion about which emotions they were experiencing, with all this resulting in them using numerous strategies to manage the emotions they experienced (Fox, 2009). These strategies will be considered later on in the synthesis under the “management of emotions” section. This finding also backs up evidence suggesting that individuals with EDs often display high levels of alexithymia and that they may be using their ED to manage emotional difficulties they experience (see Nowakowski, McFarlane, & Cassin, 2013). The following quote demonstrates this lack of ability to express emotions.

I suppose I was just sort of didn't have any emotions about, well I did, but I never showed them, never, I kind of masked it all and brushed it under the carpet …. (Fox, 2009, p. 249)

#### 3.2.2 Poor emotional bonds

This subtheme indicates why individuals with EDs came to develop such poor emotional skills, including the experience of emotions as overwhelming. Early life experiences and the poor emotional environments experienced by participants may have influenced this (Fox, 2009; Williams & Reid, 2012). Some participants described how there was very little or a complete lack of emotional expression in their families, whilst they were growing up. This was due to their caregivers' lack of ability to express emotions, or due to active denial of the emotions, which resulted in emotional confusion. By contrast, other participants described how many negative emotions existed in their childhood, particularly the emotions of anger and sadness (Fox, 2009). They often felt frightened and overwhelmed due to the experience of living with someone who was unpredictable and displaying “Jekyll and Hyde” anger, as well as frequently experiencing others displaying destructive behaviour due to feelings of anger. This contrast in the emotional environments experienced by participants is demonstrated below.

… I think he has been really sad about like how I'm feeling upset about it, and I don't know, but he kind of never showed it, he's just silent, but I know what that means that he just doesn't know what to say and that he's upset and …. (Fox, 2009, p. 287)

It was very tense around my mum and dad, especially my dad. He was very jealous, very aggressive and you never knew what to expect. I was sometimes scared to come home from school and find out what mood he was in. (Fox, 2009, p. 284)

### 3.3 Theme 2: Interpersonal vulnerability

This theme illustrates how individuals with EDs felt vulnerable in social and interpersonal interactions with family members and peers. It summarizes how they felt vulnerable in interpersonal relationships and were scared of developing social, interpersonal, and intimate relationships with others due to the negative emotions they associated with them and the high achievement expectations they believed such relationships demanded. It is to be noted that this theme is thought to be associated with the first theme, in which the development of poor emotional skills and bonds amongst individuals with ED affects social and interpersonal interaction skills with family members, peers, and partners. This theme includes two subthemes: (a) poor socioemotional bonds and (b) the anorexic voice.

#### 3.3.1 Poor socioemotional bonds

The data illustrated how individuals with EDs had a lack of social interactions and interpersonal relationships, including peer and intimate relationships (Kyriacou et al., 2009; Wasson, 2003). This subtheme demonstrates how such individuals developed a fear for social interactions and the emotions associated in interpersonal relationships due to their previous negative experiences, whilst growing up and their lack of ability to manage their emotions. The difficulties participants experienced in managing their emotions resulted in difficulties with interpersonal interactions, and as a result, they isolated themselves due to their concerns about what others thought about them (Koruth et al., 2012; Kyriacou et al., 2009; Wasson, 2003). In other words, social interactions were considered to be threatening and something to be avoided due to fear and the negative emotions such individuals associated with these relationships, including relationship problems, pressures, and high achievement expectations from family and peers (Koruth et al., 2012; Nordbo et al., 2006; Wasson, 2003; Williams & Reid, 2012). Focusing on food‐ and weight‐related issues made it difficult to find the time and energy to socialize, and subsequently, this helped with avoiding the negativity associated with these interpersonal interactions (Nordbo et al., 2006).

I might get close to someone. They might ask me to do something with them, take a walk, talk on the phone, be their friend. Then I get afraid and think I don't want a friend. I want to be alone. (Wasson, 2003, p. 83)

Things were chaotic, the job, the school, all my relationships … It's quite related to the eating disorder I guess, but I thought that as long as I had the eating disorder, I didn't need to focus on those things. Then I was thinking of food instead. (Nordbo et al., 2006, p. 559)

#### 3.3.2 The “anorexic voice”

Participants with EDs (and specifically AN) described how an internal phenomenological voice developed in the form of an inner voice on their thought processes that helped them to manage their painful emotions (Tierney & Fox, 2010; Williams & Reid, 2012). This voice filled a void, whereby it offered support in decision making, a feeling of security, and comfort when confronted with negative emotions (Tierney & Fox, 2010; Williams & Reid, 2012). In other words, the voice offered the support, which participants failed to receive from other individuals due to their lack of social skills and the negative emotions they associated with interpersonal relationships. As such, participants originally described this inner voice as a positive phenomenon, and they considered it to be a “friend” who supported them with their day‐to‐day living needs. It therefore provided them with comfort and security.

This voice was like my new life coach and I couldn't think of any reason not to listen to it. (Tierney & Fox, 2010, p. 247)

However, over time and as participants' eating behaviours progressed from what was originally considered regular dieting to a proper ED, the voice that was originally perceived as a “friend” became more dominant, controlling and a separate phenomenon within the self and no longer supported their sense of self (Tierney & Fox, 2010; Williams & Reid, 2012). Therefore, the inner voice became an entity, which was disliked by the individual. It gained control by creating feelings of shame, guilt, worthlessness, and fear within the individual if they did not obey it and feelings of insecurity with family and friends; thus, attaining a sole relationship with the patient (Tierney & Fox, 2010; Williams & Reid, 2012). Only when participants came to the realization that the inner voice denied them of everything it promised did they find the power to fight it (see Tierney & Fox, 2010; Williams & Reid, 2012).

It turned me against people. It would tell me: “They hate you and you can't really trust them. They'll only hurt you. You don't need them.” Or it would whisper: “You're evil and don't deserve them – being close to and talking to them will only hurt them. The only thing you can do right is to lose another stone. (Tierney & Fox, 2010, p. 248)

You may use it to seek perfection or love or competence or admiration or confidence … But these are the very things it takes away …. (Williams & Reid, 2012, p. 809)

### 3.4 Theme 3: The experience of negative emotions in social contexts

The results revealed that participants with EDs frequently experienced a range of negative emotions including anger, sadness, disgust, fear, shame, and guilt. However, they often failed to express these emotions due to the negative social implications they believed this could have on themselves or others, including the potential for it to cause rejection by others or cause harm to others. The emotions of shame and guilt have received more consideration in the qualitative ED literature, with there having been a particular focus of the role of shame and guilt amongst mothers with an ED (Espeset et al., 2012; Jeppson et al., 2003; Rortveit et al., 2010; Rortveit, Astrom, & Severinsson, 2009; Skarderud, 2007). Therefore, this overarching theme is made up of the following two subthemes: (a) negative emotional beliefs and (b) shame and guilt as a mother. These subthemes demonstrate that whilst individuals with EDs were worried about the negative interpersonal repercussions that expressing these emotions would have, interpersonal and social factors also aroused these emotions; for example, being a mother with an ED triggered feelings of shame and guilt, which will be elaborated on below.

#### 3.4.1 Negative emotional beliefs

Many participants discussed how they experienced negative emotions most of the time but were afraid to express them partly due to their belief that they lacked the skills to do so, but largely due to the negative interpersonal implications, they believed it would have. Similarly, they discussed how they would avoid certain social situations, which could trigger some of these negative emotions. These emotions included those of anger, sadness, fear, disgust, and shame (Espeset et al., 2012; Fox, 2009; Jeppson et al., 2003;Rortveit et al., 2010 ; Skarderud, 2007).

The emotion of anger was described by participants as one of the most challenging to manage (Espeset et al., 2012; Fox, 2009). This was due to ED patients thinking it was a dangerous emotion on both an interpersonal and intrapersonal level. They were worried that they would lose control of themselves or that it could cause them to be rejected by others (Espeset et al., 2012; Fox, 2009). Sadness was described as another one of the most difficult of emotions to experience, and participants mentioned how they spent most days feeling sad and lonely but felt they were not able to share this with others (Espeset et al., 2012; Fox, 2009). This was due to the beliefs that expressing sadness demonstrated weakness resulting in an increased sense of vulnerability to others. For others, expressing sadness was often perceived as potentially harmful (Espeset et al., 2012; Fox, 2009).

I was never angry! Still, I don't get angry. Perhaps I get irritated, but I don't get angry. I think it's terrifying to become angry. It's unpredictable and scary. What will actually happen if I become angry? Maybe they will never talk with me again. (Espeset et al., 2012, p. 456)

In relation to fear, participants described how they experienced this emotion most of the time as part of their ED (Espeset et al., 2012; Fox, 2009). They felt worried about weight gain and becoming fat, losing control of their eating, and they were nervous about what others thought about them and their appearance and body. Therefore, this emotion was generally aroused via interpersonal interactions. Moreover, disgust was described by participants as an invasive emotion and aroused by eating, looking in the mirror, or having their bodies commented on by others, which resulted in them thinking they were overweight and disgusting (Espeset et al., 2012; Fox, 2009). Therefore, this emotion was also aroused via feelings of social vulnerability and threat, and participants avoided experiencing disgust by avoiding social interactions. This social vulnerability seems to be related to the previous theme elaborated on, namely, “interpersonal vulnerability” and “poor socioemotional bonds,” in which the data demonstrated how individuals with EDs felt vulnerable in social and interpersonal interactions due to the negative emotions and high expectations they associated with them. For these reasons, such individuals attempted to avoid social and interpersonal interactions, which aroused negative emotions. The following quote illustrates the fear and disgust aroused by social interactions amongst patients with EDs.

I'm so concerned about what others think about me when they meet me. I'm so afraid that they think that I'm too fat. And when I come to the meeting place, I get so nervous. I just feel fat and disgusting. (Espeset et al., 2012, p. 457)

The feeling of shame and unworthiness was aroused by both internal factors and external demands (Espeset et al., 2012; Jeppson et al., 2003; Rortveit et al., 2010; Skarderud, 2007), including their negative emotions, failure to achieve goals or demands made by other people, lack of control over ones behaviours, abuse by others, and their ED (Rance et al., 2017; Rortveit et al., 2010; Skarderud, 2007). It appears that the “body” becomes used as means of expressing and controlling negative emotions through the use of food control (Skarderud, 2007). This ability to lose weight was found to produce a sense of pride amongst patients, which served as a maintenance factor for AN (Faija et al., 2017). Therefore, it appears that interpersonal issues such as the experience of shame become more of an intrapersonal issue (Rortveit et al., 2010; Skarderud, 2007).

I feel ashamed about everything. I feel ashamed about feeling ashamed. (Skarderud, 2007, p. 86)

#### 3.4.2 Shame and guilt as a mother

Two studies specifically reported on the experiences of guilt and shame amongst individuals with an ED who were also mothers (Rortveit et al., 2010; Rortveit, Astrom, & Severinsson, 2009). The feelings of guilt and shame appeared to be intertwined amongst mothers with EDs (Rortveit et al., 2010). Mothers with EDs reported shame and guilt in relation to concerns about their adequacy as a mother and concerns about the impact of their ED on their children. In relation to concerns about their role as a mother, participants reported feeling guilty about the fact that their weakened state due to their illness had impacted on their relationship with their children (Rortveit, Astrom, & Severinsson, 2009). Mothers also reported guilt and shame about the effect their ED may have on their children (Rortveit et al., 2010; Rortveit, Astrom, & Severinsson, 2009). As a result, they tried to hide their illness as they did not want their children to develop it or to blame themselves for their mother's illness. This made normal routine activities such as mealtimes difficult as they could not join in with them. Finding the right balance of information to give to their children about their ED was difficult, because no information would leave them suspicious but too much information could harm them as well.

I couldn't attend it [daughter's wedding] because I was too “entrapped in my own system”. In a way I was too unwell to become involved. As a matter of fact, I thought it was a burden at the start. And everybody else was very happy … Lots of feelings and many expectations … But it was the opposite for me … There is still too much in my head to take it all in. (Rortveit, Astrom, & Severinsson, 2009, p. 606)

I am afraid that they will be exposed to … .That they will develop eating problems themselves …. (Rortveit et al., 2010, p. 235)

### 3.5 Theme 4: Management of emotions

This overarching theme is considered to be a result of an interplay between all previous themes highlighted in this review. This theme highlights how participants with EDs struggled to manage the emotions they experienced and describes how they used various strategies including their ED symptoms to avoid having to deal with these emotions. This inability to handle emotions in a constructive way is not surprising considering the development of poor emotional skills, difficulty with interpersonal interactions and the negative emotions experienced by participants, which have all been elaborated on in the previous sections of the results. Two subthemes that emerged from the data were (a) internal emotional control (distraction, emotional suppression, and releasing emotions), which involved individuals with EDs using the disorder and internal or personal strategies to manage their emotions and (b) external emotional control (inhibition of emotions and external avoidance), which involved these individuals using external social and interpersonal strategies for managing and regulating their emotions.

#### 3.5.1 Internal emotional control

Participants described how they used their ED as a general means of managing and controlling their emotions (Fox, 2009; McNamara et al., 2008; Rortveit, Vevatne, & Severinsson, 2009). Participants explained how having an ED and engaging in behaviours related to their ED helped them manage their emotions and that gaining control of their ED and not engaging in ED‐related behaviours resulted in loss of control of emotions (Kyriacou et al., 2009; Rortveit, Vevatne, & Severinsson, 2009b). They experienced positive emotions when in control of their eating behaviour and negative emotions when they lost control of their eating (Jeppson et al., 2003; Williams & Reid, 2012).

Previously I used to compensate by purging … But now that I no longer do it, so many feelings come up. I have to deal with them in other ways. (Rortveit, Vevatne & Severinsson, 2009, p. 321)

Control … it made me feel empowered. (Jeppson et al., 2003, p. 118)

The data further revealed how participants used three specific internal or intrapersonal emotion regulation strategies and behaviours related to their ED to manage the negative emotions they experienced (Espeset et al., 2012; Fox, 2009; Jeppson et al., 2003; Nordbo et al., 2006; Williams & Reid, 2012). Distraction was a strategy described by participants, which involved them using their concerns about eating and gaining weight to distract themselves and take their focus away from any negative emotions they experienced, particularly sadness, anger, and disgust (Espeset et al., 2012; Nordbo et al., 2006; Williams & Reid, 2012). According to Gross (1998), distraction is actually an adaptive antecedent‐focused emotion regulation strategy preventing emotional arousal in the first place.

Food was all I could think about. I used it to comfort myself and run away from my life. (Williams & Reid, 2012, p. 804)

I think it was a way to change focus. Because when I focus on exercising, eating healthy and losing weight, there's no room for thinking about being sad. (Espeset et al., 2012, p. 455)

Emotional suppression involved using ED behaviours and symptoms including food restriction, purging, and physical activity to control the degree to which they experienced their negative emotions or to stop experiencing them altogether (Espeset et al., 2012; Jeppson et al., 2003). This was due to their ability to stop or weaken the intensity of emotional responses and was frequently used to suppress the emotions of sadness and fear (Espeset et al., 2012). In addition, participants described how their AN, which resulted in weight loss, caused a “numbing effect” (Fox, 2009; Kyriacou et al., 2009; Williams & Reid, 2012). The third strategy was that of releasing emotions. It involves using ED behaviours, for example, food restriction, self‐control, or self‐harm to express negative emotions (Espeset et al., 2012). The main emotion for which this was used was for that of anger, which participants sometimes found uncontrollable and needed to be released.

I cut myself if I want to punish myself. It's when I want myself to feel pain and if I feel that I'm stupid or incapable or like I'm not worth anything. I also use laxatives to punish myself. But it's really painful. If you take hundreds of laxatives, you really think that you're gonna die. (Espeset et al., 2012, p. 456)

#### 3.5.2 External emotional control

The data also demonstrated how participants used emotion regulation strategies, which involved external and interpersonal means of controlling their emotions. Two strategies used were those of inhibition of emotions and the external avoidance of emotions. Inhibition of emotions was a strategy used by participants, which involved them failing to express their emotions when surrounded by others (Espeset et al., 2012; Fox, 2009). Participants said how they frequently felt sad and depressed but hid this from others (Espeset et al., 2012). Moreover, participants would engage in physical activity when feeling angry in order to avoid having to confront their anger (Espeset et al., 2012). The following statement illustrates this emotion inhibition.

I just see it, if I start crying it's a sign of weakness and something that I don't like doing really. (Fox, 2009, p. 293)

External avoidance of emotions involved participants avoiding any environments or social interactions, which could arouse these emotions, including sexual relationships and social interactions involving eating (Espeset et al., 2012; Nordbo et al., 2006; Williams & Reid, 2012). One emotion that was managed using external avoidance was that of disgust (Espeset et al., 2012).

I don't like being touched, and especially not my stomach. Because then I feel fat. I think it's disgusting …. (Espeset et al., 2012, p. 457)

It is to be noted that the two external emotion regulation strategies highlighted above, which demonstrated how participants managed their negative emotions either by failing to express them in front of others or by avoiding the social contexts which may arouse them, provides further evidence for the interpersonal vulnerability participants believed would result from expressing negative emotions, which has been described earlier on in the synthesis.

### 3.6 The emotional self within a social context

This is a third‐order theme, which emerged from the second‐order themes, and goes beyond the original data. The model we developed demonstrates a process between the individual with an ED in a social environment and how environmental experiences influence how they experience and manage emotions. Throughout the synthesis, the themes demonstrate how social factors including poor emotional environments and development whilst growing up influences how these individuals experience and express negative emotions, as well as resulting in the development of poor socioemotional bonds. Subsequently, this poor socioemotional development results in maladaptive interpersonal and intrapersonal strategies involving ED characteristics being used to manage negative emotions and perceived negative socioemotional interactions.

## 4 DISCUSSION

The aim of this review was to gain further insight into the role of emotions in EDs by conducting a metasynthesis of qualitative studies. The metasynthesis consisted of 16 studies involving a total of 203 participants, with 69% presenting with AN. We identified four second‐order themes and one third‐order theme, with the second‐order themes consisting of numerous subthemes (see Figure 2). The second‐order themes are those of negative emotional environments, interpersonal vulnerability, the experience of negative emotions in social contexts, and management of emotions, whilst the third‐order theme is that of “the emotional self within a social context.” A model was developed to illustrate how all these themes interact and influence each other in individuals with EDs.

In relation to the second‐order themes, the theme of “negative emotional environments” revealed how individuals with EDs developed poor emotional skills as a result of early childhood experiences. The review identified that they experienced problems with emotional awareness, emotional expression, and experiencing emotions as overwhelming. These findings are in line with the SPAARS‐ED (Fox & Power, 2009; Power & Dalgleish, 2008), which suggests that an individual's emotional reactions are a result of their past experiences in terms of their family environment, attachment patterns, and experiences of abuse (Bruch, 1973; Corstorphine, 2006; Espina, 2003; Kent & Waller, 2000; Kyriacou, Treasure, & Schmit, 2008; Smolak & Levine, 2007; Ward et al., 2001; Ward, Ramsay, & Treasure, 2000). They are also in line with the INTEGRATE‐AN model (Hatch et al., 2010) in that the model suggests that negative life experiences along with emotional sensitivity results in a feeling of loss of control and that ED symptoms are used to regulate these negative emotions. The fact that the review highlighted that individuals with EDs struggled to express their emotions largely due to the negative beliefs they held about expressing negative emotions further supports the SPAARS model (see Fox & Power, 2009). It further supports studies, which found that individuals with EDs experience high levels of negative emotions, and particularly anger and sadness, but fail to express these emotions (see Fox & Froom, 2009; Fox & Harrison, 2008; Geller, Cockell, Hewitt, Goldner, & Flett, 2000; Waller et al., 2003; Waller, Corstorphine, & Mountford, 2007).

A second theme highlighted by this review was “interpersonal vulnerability,” which related to the difficulties with interpersonal interactions involving friends, family, and partners individuals with EDs displayed. They often tried to avoid such social interactions due to the negative emotions they associated with these relationships and used their ED symptoms to do so. It was due to this social void that the “anorexic voice” developed offering support in decision making, feelings of security, and comfort when confronted with negative emotions (Tierney & Fox, 2010; Williams & Reid, 2012). These findings provide further support for the cognitive–interpersonal maintenance model of AN (Schmidt & Treasure, 2006; Treasure & Schmidt, 2013), which suggests that individuals with EDs engage in emotional avoidance through the avoidance of social interactions and that the presence of the ED further exacerbates these social and emotional skills. They also support the INTEGRATE‐AN model (Hatch et al., 2010), which suggests that such individuals are more sensitive to negative emotional cues. The present findings of the negative emotions individuals associated with such social and interpersonal interactions also backs up the quantitative evidence showing that ED patients demonstrate attentional bias to negative but not positive facial expressions (Cardi, Matteo, Corfield, & Treasure, 2013; Cserjesi, Vermeulen, Lenard, & Luminet, 2011; Harrison, Sullivan, Tchanturia, & Treasure, 2010), show difficulty in recognizing the emotions of others (Jansch, Harmer, & Cooper, 2009; Oldershaw et al., 2011), and show difficulty in expressing their own emotions (Claes et al., 2012; Davies, Schmidt, Stahl, & Tchanturia, 2011; Jansch et al., 2009), which are all important factors for interpersonal communication (see Treasure & Schmidt, 2013).

The third main theme highlighted was “the experience of negative emotions in social contexts” and related to the general negative emotions experienced by the individuals with EDs and how they failed to express them in social contexts. The emotions experienced included anger, sadness, fear, disgust, and shame, as well as the experience of shame and guilt in mothers with EDs. The experience of lots of negative emotions amongst these individuals is suggested by the INTEGRATE‐AN model (Hatch et al., 2010). In addition, this finding provides support for the SPAARS model, which suggests that individuals with EDs suppress their emotions using their ED and therefore are directing them towards their body (Fox & Power, 2009). In addition, the emotions of shame and guilt may be more pertinent for mothers with EDs. Further qualitative and quantitative research is required to clarify the range of negative emotions, which may be experienced by these patients, and whether the experience of them is also dependent on one's status, for example, being a mother.

The final theme highlighted by this review was that of the “management of emotions.” This theme was considered to be the result of an interplay between the previous three themes and involved individuals using internal or external aspects of their ED to manage negative emotions. These findings provide further support for and add to the models of emotion regulation, which have been referred to in this review (see Fox & Power, 2009; Gross, 1998, 2002;Schmidt & Treasure, 2006 ; Treasure & Schmidt, 2013). The SPAARS‐ED model (Fox & Power, 2009), cognitive–interpersonal maintenance model (Schmidt & Treasure, 2006; Treasure & Schmidt, 2013), and INTEGRATE‐AN (Hatch et al., 2010) all suggest that ED behaviours are used to manage and regulate emotions. The current review adds to these models in that it elaborates on how the different negative emotions may be regulated using different ED behaviours and emotion regulation strategies (e.g., Espeset et al., 2012).

The third‐order theme of “the emotional self within a social context” demonstrates how amongst individuals with EDs emotional perception, expression of emotions, and emotional responses are influenced by social and interpersonal factors. The experience of poor emotional environments whilst growing up influences how individuals with EDs perceive and manage negative emotions within social or interpersonal contexts and results in the possible maladaptive internal and external strategies being used to regulate them. These maladaptive strategies are frequently symptoms and behaviours relating to an ED, including the avoidance of interpersonal interactions that arouse emotions. This theme builds on the cognitive–interpersonal maintenance model of AN (Schmidt & Treasure, 2006; Treasure & Schmidt, 2013) and the INTEGRATE‐AN model (Hatch et al., 2010), both suggesting that EDs are a result of an interaction between social and emotional factors with a need to control and regulate negative emotions.

### 4.1 Practical and clinical implications

The findings of this review have a number of important practical and clinical implications. The most significant factor to highlight is that an ED appears to help individuals manage their emotions (e.g., Nordbo et al., 2006; Williams & Reid, 2012). Thus, the central part of any treatment should involve learning effective strategies to manage and control one's emotions. The SPAARS‐ED model, dialectical behaviour therapy, and emotion‐focused therapy (see Fox, Federici, & Power, 2012b) focus on emotions and teach effective strategies to manage them. However, this paper highlights how other factors need to be included in interventions. For example, our model demonstrates how certain social and familial factors may make an individual at greater risk for EDs, but that these weaknesses are further attenuated by the ED and that treatment needs to be comprehensive and involve the patients and significant others (Treasure & Schmidt, 2013). The model suggests that an individual's past emotional environment, ability to manage emotions, interpersonal relationships, and social role (e.g., being a mother) need to be considered (Fox, 2009; Koruth et al., 2012; Kyriacou et al., 2009; Rortveit et al., 2010; Rortveit, Astrom, & Severinsson, 2009; Wasson, 2003). It is also possible that some behavioural cycles and maintenance factors found in EDs may have become divorced from these earlier precipitating social and emotional factors (e.g., Cooper, Wells, & Todd, 2004). Thus, the treatment process must take into account all the different social and interpersonal factors of an individual's situation, in addition to providing emotional skills training.

### 4.2 Limitations of the present review

Based on the appraisal system used in this review (CASP, 2006; Walsh & Downe, 2006) and drawing on additional guidelines for the publication of qualitative research (Elliot, Fischer, & Rennie, 1999), three limitations regarding the quality of the studies in this review can be highlighted. The first issue relates to the sampling methods utilized by the studies. All but two of the studies relied on clinical assessments for identifying EDs or the fact that participants attended ED self‐help organizations and failed to assess for these disorders for the purpose of the research. Second, the majority of the authors failed to describe their study's theoretical backgrounds and how this may have influenced the research process or vice versa. Finally, most studies failed to provide any information on the context of the research and data collection, which may have influenced the findings and their transferability.

This review also did not differentiate between the different EDs. This was due to the limited number of studies on emotions and EDs and that the goal of this review was to consider generally the role of emotions in EDs, which was also characteristic of some of the studies included (e.g., Espeset et al., 2012). Moreover, much of the literature pertaining to emotions and EDs discusses EDs in general (see Fox et al., 2012a), possibly because EDNOS is more prevalent in patients with EDs. Most patients do not fully meet the diagnostic criteria for AN or BN (Crow, 2007; Fairburn et al., 2007; Fairburn, Cooper, & Shafran, 2003; Fox et al., 2012a; Walsh & Sysko, 2009), with some patients' ED diagnosis changing over time (Milos, Spindler, Schnyder, & Fairburn, 2005). However, a higher percentage of participants presented with AN in this review (69%) and this needs to be taken into account. There is some research suggesting that emotional processing and regulation is more severely disrupted in AN than BN, with individuals with AN demonstrating more difficulties with basic emotion processing and expression and individuals with BN having more difficulties with self‐conscious emotions (e.g., shame and impulsivity; Forbush & Watson, 2006; Hayaki, Friedman, & Brownell, 2002). However, further research is required to clarify emotional differences in these disorders (Pascual, Etxebarria, & Cruz, 2011).

### 4.3 Future research

Further research is needed to validate the findings of this review. Longitudinal research is required to confirm the temporal sequence on the relationship between negative social environments and the development of poor social and emotional skills. The model in this review suggests that the experience of negative emotional environments, whilst growing up, results in the development of poor social and emotional skills amongst individuals with ED, but it may be that these negative environments are in part a result of having the presence of an individual with an ED within the family. The relationship between negative emotional environments, poor emotional skills, and having an ED may also be reciprocal (see Corstorphine, 2006; Fox et al., 2012a). In addition, research is required to further validate the finding that individuals with ED use different emotion regulation strategies for the different negative emotions they may experience (Espeset et al., 2012) and whether the emotions experienced and how they are managed vary depending on an individual's role and responsibility, for example, being a mother (Rortveit et al., 2010). It would also be useful to consider whether any of the findings presented in this review vary as a function of the different subtypes of EDs (Skarderud, 2007).

## 5 CONCLUSION

In conclusion, we conducted the first metasynthesis of qualitative studies considering emotions in EDs in order to gain further insight into the role emotions play in ED with a view to refine psychological interventions. Our model provides further support for the SPAARS‐ED model (Fox & Power, 2009), the cognitive–interpersonal maintenance model (Schmidt & Treasure, 2006; Treasure & Schmidt, 2013) and the INTEGRATE‐AN model (Hatch et al., 2010). However, the finding that individuals with EDs use their disorder and associated symptoms to manage the negative emotions they experience in interpersonal and intrapersonal situations differently is novel.
